# Protocol for the safety of brodalumab regarding psychiatric comorbidities in patients with psoriasis

**DOI:** 10.1192/j.eurpsy.2022.1957

**Published:** 2022-09-01

**Authors:** O. Pikou, P. Argitis, A. Karampas, S. Karavia, Z. Chaviaras

**Affiliations:** 1 General Hospital of Corfu, Dermatology, corfu, Greece; 2 General Hospital of Corfu, Psychiatric, Corfu, Greece

**Keywords:** psoriasis, il-17

## Abstract

**Introduction:**

Brodalumab, an anti-IL17RA monoclonal antibody, is a treatment option for plaque psoriasis related to increased risk of suicidal ideation and behavior, including completed suicide.However, in the literature no causal relationship is reported, and the reported incidence of patients receiving brodalumab that committed suicide did not differ from the expected suicide incidence of the general population.

**Objectives:**

Our objective is to answer the followings:1) What is the probability of developing emotional disorders during treatment with brodalumab.2) What is the recurrence rate of previously diagnosed emotional disorders in brodalumab responders (Kaplan-Meier curve). 3) What is the relationship between suicide and/or suicidal ideation in patients receiving treatment with brodalumab.

**Methods:**

The study will enroll patients with moderate to severe psoriasis (aged 18-70) that attend the outpatient dermatology, and who already receive or are eligible to receive treatment with brodalumab. Patients who are willing to participate in the study will be provided with the self-completing questionnaires PHQ-9, GAD-7, RASS, HRQoL. During follow-up, if a patient presents with a PHQ> 9, will be referred for an HDRS evaluation in which if score> 17 in at least 2 scores or an increase in the RASS scale is detected, will be referred for psychiatric intervention.

**Results:**

Assessments will take place a) before treatment , b) 2 weeks after initiation of treatment with brodalumab , c) every month for the first 3 months, and e) on a quarterly basis up to 52 weeks.

**Conclusions:**

This study will focus on creating a protocol for the safe use of brodalumab in psoriasis patients with concomitant psychological/psychiatric comorbidities.

**Disclosure:**

No significant relationships.

